# Quality of life of X-linked agammaglobulinemia patients in the United Kingdom

**DOI:** 10.70962/jhi.20250198

**Published:** 2026-02-19

**Authors:** Ben Shillitoe, Helen Bourne, Catherine Stroud, Terry Flood, Matthew Buckland, Winnie Ip, Austen Worth, Scott Hackett, Archana Herwadkar, Tanya Coulter, Stephen Jolles, Tomaz Garcez, Eduardo Moya, Saul N. Faust, Mark S. Pearce, Andrew R. Gennery

**Affiliations:** 1 Sheffield Children’s NHS Foundation Trust, Sheffield, UK; 2 Royal Victoria Infirmary, Newcastle upon Tyne, UK; 3 Great North Children’s Hospital, Newcastle upon Tyne, UK; 4 Institute of Child Health, UCL, London, UK; 5 Department of Immunology and Gene Therapy, Great Ormond Street Hospital for Children NHS Foundation Trust, London, UK; 6 University Hospitals Birmingham NHS Foundation Trust, Birmingham, UK; 7 Salford Care Organisation, Northern Care Alliance NHS Trust, Salford, UK; 8 Belfast Health and Social Care Trust, Belfast, UK; 9 Immunodeficiency Centre for Wales, University Hospital of Wales, Cardiff, UK; 10 Manchester University NHS Foundation Trust, Manchester, UK; 11 Bradford Teaching Hospitals NHS Foundation Trust, Bradford, UK; 12 NIHR Southampton Clinical Research Facility and Biomedical Research Centre, University Hospital Southampton NHS Foundation Trust, Southhampton, UK; 13 Faculty of Medicine and Institute for Life Sciences, University of Southampton, Southhampton, UK; 14 Population Health Sciences Institute, Newcastle University, Newcastle, UK; 15 Newcastle University Translational and Clinical Research Institute, Newcastle University, Newcastle, UK

## Abstract

Due to limitations in current therapies (immunoglobulin replacement therapy), complications in X-linked agammaglobulinemia (XLA) such as bronchiectasis may continue to occur, with subsequent significant impacts on health-related quality of life (HRQoL). There were no significant differences in PedsQL 4.0 total scores against UK healthy norms (self 80.98, P = 0.277; parent 79.76, P = 0.465). There were no significant differences in the SF36v2 physical component score (PCS) against UK healthy norms (49.10, P = 0.712). However, XLA patients with bronchiectasis had significantly worse PCS than patients without (47.88 versus 55.14, P < 0.001) and significantly worse PCS than UK healthy norms (P = 0.004). In the absence of bronchiectasis, HRQoL is comparable to UK healthy norms. However, XLA patients with bronchiectasis have significantly worse HRQoL than patients without bronchiectasis and UK healthy norms. These data demonstrate that further work into novel therapies are needed to prevent bronchiectasis to enable XLA patients to have a normal quality of life.

## Teaser

Assessment of UK XLA patients reveals that bronchiectasis, present in the majority of adults, is strongly associated with diminished quality of life. Without lung disease, patients experience largely normal QoL, emphasizing the critical role of respiratory health in outcomes.

## Introduction

The discovery of X-linked agammaglobulinemia (XLA) by Bruton in 1952, and the subsequent treatment using immunoglobulin replacement therapy (IgRT) (1), is often cited as the birth of clinical immunology (2). XLA is caused by defects in the Bruton tyrosine kinase (*BTK*) gene, causing B lymphocyte maturation arrest at the pro-B to pre-B developmental stage, a significant reduction in circulating CD19^+^ B lymphocytes and subsequent agammaglobulinemia (3, 4, 5, 6, 7, 8, 9).

Significant improvements in IgRT have been made via the development of modern subcutaneous (SC) and intravenous (IV) preparations in the 1980s, which allowed longer-term survival into adulthood. However, the foundation of treatment has not changed in over 70 years (1, 10, 11).

Despite IgRT, complications of XLA may still occur. Plebani et al. reported chronic lung disease in 33% of their cohort (24/73) in 2002, with a calculated 25 years post-diagnosis risk of developing chronic lung disease of 92% (12). Bronchiectasis may be due to the lost BTK functions beyond B lymphocyte development or to the lack of IgA and IgM replacement, which play important roles in protection of mucosal surfaces (13, 14, 15, 16, 17).

With these limitations in mind, there are potential advances in management, including newborn screening by measurement of κ-deleting recombination excision circles, curative hemopoietic stem cell transplantation (HSCT), and gene therapy (18, 19, 20). None of these strategies have entered routine clinical practice, requiring more accurate assessment of current XLA outcomes, including quality of life (QoL).

Health-related QoL (HRQoL) is an important tool in evaluating outcomes in both clinical practice and research. HRQoL has been used in evaluating outcomes of treatment for inborn errors of immunity (IEI) and has aided greatly in evaluating the role of differing management strategies for clinicians, families, and patients alike (21, 22). Previous work has reported conflicting information on whether or not HRQoL in XLA is comparable to the United Kingdom (UK) norms (23, 24). Like other IEI, XLA is an extremely rare disorder, with a UK prevalence of 2.9 cases per 1 million population (25). Existing studies are limited by small sample sizes and limited information to correlate HRQoL with clinical outcomes.

This study aimed to describe the HRQoL of XLA patients in the UK and correlate this with clinical outcomes to help identify potential areas of improvement or development in the care of XLA patients.

## Results

Selected clinical and demographic data taken from our study assessing clinic outcomes are shown in Table 1 (26). Of these 76 eligible patients, 67 patients completed the HRQoL assessments, representing an overall return rate of 88% (Table 1). The return rate was high among adults, with 44 of 48 eligible patients participating (92%), while 23 of 28 eligible pediatric patients participated (82%). While the majority of participants completed all survey domains, the specific number of respondents (*n*) for each clinical measure and QoL instrument is reported in the corresponding tables to ensure transparency regarding data attainment. 4% of the pediatric cohorts have evidence of bronchiectasis on their latest high-resolution computed tomography (HRCT), with 64% of the adult cohort showing evidence of bronchiectasis.

**Table 1.** Demographic data for the cohort

​	
**Pediatric cohort**	
Cohort size	23 (82% response rate)
Age (years)	7.81 (3.90, 12.49)
Age at diagnosis (years) (*n* = 20)	1.19 (0.80, 2.93)
Screened due to family history	6 (21%)
Home IgRT	16 (70%)
*Home SCIg*	15 (94%)
Bronchiectasis	1 (4%)
Conjunctivitis	2 (9%)
Post-infection deafness	1 (4%)
Infective gastrointestinal complications	1 (4%)
Noninfectious gastrointestinal complications	1 (4%)
Mental health disorder	1 (4%)
Median annual bacterial infection incidence (*n* = 20)	1.20 (0.80, 1.88)
Last recorded FEV1 Z-score (*n* = 10)	0.21 (−1.02, 0.60)
**Adult cohort**	
Cohort size	44 (92% response rate)
Age (years)	36.22 (29.15, 48.76)
Age at diagnosis (years) (*n* = 39)	3.37 (1.16, 10.79)
Screened due to family history	12 (25%)
Home IgRT	31 (74%)
*Home SCIg*	14 (45%)
Bronchiectasis	28 (64%)
Conjunctivitis	7 (16%)
Post-infection deafness	3 (7%)
Infective gastrointestinal complications	1 (2%)
Noninfectious gastrointestinal complications	4 (9%)
Mental health disorder	9 (21%)
Median annual bacterial infection incidence	0.91 (0.68, 1.50)
Last recorded FEV1 Z-score	−1.23 (−3.00, −0.65)

Data are shown as median (IQR). The denominator is shown where not all data are available. SCIg, SC immunoglobulin.

While respiratory complications predominated, other clinical comorbidities were recorded. In the adult cohort (*n* = 44), these included conjunctivitis (16%), mental health disorders (21%), and noninfectious gastrointestinal complications (9%) (Table 1). Post-infection deafness was present in 7% of adults. Infectious gastrointestinal complications were less frequent, affecting only 2% of the adult cohort. No patients in the study had a diagnosis of nodular regenerative hyperplasia or chronic enteroviral meningoencephalitis.

### Pediatrics

#### Paediatric Quality of Life Inventory (PedsQL) 4.0

A total of 16 children and 23 parents filled out the PedsQL 4.0 (Table 2).

**Table 2.** PedsQL 4.0 scores (median, IQR) versus UK norms (mean, SD) (27) and CF patients (28) (mean, SD)

​	UK cohort	UK norms	P value	CF patients	P value
**Self-scores (** ***n*** ** = 16)**					
Psychosocial score	76.67 (67.50–83.33)	80.50 (14.06)	0.134	72.3 (1.47)	0.326
Physical score	87.50 (83.26–92.19)	86.08 (14.06)	0.796	79.2 (1.64)	0.062
Emotional score	67.50 (60.00–80.00)	76.99 (18.43)	0.062	70.7 (1.92)	0.407
Social score	100.00 (80.00–100.00)	86.85 (16.86)	0.598	78.8 (1.73)	0.073
School score	70.00 (52.50–77.50)	77.29 (16.92)	*0.049*	65.6 (1.86)	0.836
Total score	80.98 (73.91–85.87)	82.25 (13.09)	0.277	74.7 (1.42)	0.134
**Parent scores (** ***n*** ** = 23)**					
Psychosocial score	76.67 (70.00, 84.62)	79.00 (14.70)	0.626	73.3 (1.23)	0.144
Physical score	87.50 (81.25, 90.63)	84.99 (16.08)	0.855	79.3 (1.52)	0.259
Emotional score	70.00 (60.00, 85.00)	74.67 (17.67)	0.484	68.0 (1.55)	0.563
Social score	100.00 (85.00, 100.00)	84.62 (17.24)	*0.038*	81.7 (1.53)	*0.012*
School score	70.00 (55.00, 83.33)	77.72 (18.50)	*0.024*	68.8 (1.65)	0.884
Total score	79.76 (76.19, 89.13)	81.12 (13.85)	0.465	75.3 (1.22)	0.083

Values in italics are those that are statistically significant (P <0.05).

Both parent and self-scores were largely comparable to UK norms and UK cystic fibrosis (CF) patients (Table 2). UK XLA pediatric patients did self-score significantly lower in the school score domain than UK norms. Parents of XLA pediatric patients scored significantly higher in the social score compared to both UK norms and UK CF patients. They scored significantly lower in the school score compared to UK norms.

There were no significant differences in the parent or self-reported total scores in patients receiving home versus hospital IgRT (P = 0.807 and P = 0.882, respectively). There were no significant differences in parent reported total scores for patients receiving IV versus SC IgRT (77.80 versus 80.40, P = 0.378). There were no significant differences in self-reported total scores for patients receiving IV versus SC IgRT (58.70 versus 81.50, P = 0.327).

#### Strength and difficulties questionnaire (SDQ)

A total of 11 children and 23 parents filled out the SDQ (Table 3).

**Table 3.** SDQ scores for the cohort (median, IQR) versus UK norms (mean, SD) (29)

Component	Cohort	UK norms	P value
**Self-scores (** ***n*** ** = 11)**			
Emotional score	2.00 (1.00, 4.00)	2.60 (1.9)	0.592
Conduct score	2.00 (1.00, 3.00)	2.40 (1.7)	0.372
Hyperactivity score	4.00 (3.00, 5.00)	3.90 (2.2)	0.371
Peer score	0.00 (0.00, 5.00)	1.60 (1.4)	0.928
Prosocial score	8.00 (7.00, 9.00)	7.50 (1.7)	0.717
Impact score	0.00 (0.00, 0.00)	0.30 (0.8)	0.256
Total score	10.00 (5.00, 14.00)	10.50 (5.1)	0.788
**Parent scores (** ***n*** ** = 23)**			
Emotional score	2.00 (1.00, 5.00)	1.8 (2.0)	0.393
Conduct score	1.00 (1.00, 4.00)	1.7 (1.8)	0.541
Hyperactivity score	4.00 (3.00, 6.00)	4.0 (2.7)	0.174
Peer score	0.00 (0.00, 1.00)	1.5 (1.7)	*0.013*
Prosocial score	9.00 (7.00, 9.00)	8.3 (1.6)	0.976
Impact score	0.00 (0.00, 1.00)	0.5 (1.2)	0.181
Total score	9.00 (7.00, 15.00)	9.1 (6.0)	0.542

Values in italics are those that are statistically significant (P <0.05).

SDQ scores were largely comparable to UK norms for both self-reported and parent scores, apart from parent scores for the peer domain, which were significantly lower than UK norms.

Using the SDQ score results, 21% of patients are likely to have a hyperactivity disorder. 34 % are predicted to possibly or probably have a conduct disorder and 21% to possibly or probably have an emotional disorder (Table S1).

### Adults

#### Short form 36 version 2 physical health  (SF36v2) 

A total of 42 adults filled out the SF36v2 (Table 4).

**Table 4.** SF36v2 physical health scores for the cohort and by presence of bronchiectasis (median, IQR) compared to male UK norms (30) and UK male patients with CF (31) (mean, SD)

​	UK XLA patients (*n* = 42)	UK norms	P value	CF patients	P value
Physical function	95.00 (90.00, 100.00)	89.76 (18.78)	0.062	82.4 (20.7)	*0.010*
Role—physical	96.88 (87.50, 100.00)	89.01 (21.09)	0.083	75.00 (22.5)	*0.004*
Pain	74.00 (62.00, 100.00)	81.25 (22.21)	0.335	84.2 (19.6)	0.249
General health	37.00 (22.00, 52.00)	70.86 (20.29)	*<0.001*	46.8 (24.0)	0.077
PCS	49.10 (47.14, 54.95)	50.63 (9.41)	0.712	N/A	N/A

​	Bronchiectasis	No bronchiectasis	Bronchiectasis versus no bronchiectasis	Bronchiectasis versus male UK norms	Bronchiectasis versus CF patients
Physical function	90.0 (85.00, 95.00)	100.00 (100.00, 100.00)	*<0.001*	0.515	0.311
Role—physical	93.75 (75.00, 100.00)	100.00 (100.00, 100.00)	*0.008*	0.923	0.260
Pain	74.00 (62.00, 84.00)	100.00 (100.00, 100.00)	*<0.001*	*0.005*	*0.003*
General health	25.00 (17.00, 37.00)	57.00 (52.00, 75.00)	*<0.001*	*<0.001*	*0.001*
PCS	47.88 (44.94, 50.09)	55.14 (53.30, 57.47)	*<0.001*	*0.004*	N/A

Values in italics are those that are statistically significant (P <0.05).

UK XLA patients scored significantly higher than CF patients in the physical function and physical role domains. They scored significantly lower in the general healthy domain compared to UK norms. There were no significant differences in the remaining domains.

XLA patients with bronchiectasis scored significantly lower in all domains compared to those without. XLA patients with bronchiectasis scored significantly lower in the pain and general health domains compared to both UK norms and CF patients. In addition, patients with bronchiectasis scored significantly lower in the overall physical component scores (PCS) compared to UK norms.

Increasing age correlated with a worse score in the PCS of the SF36v2, but other phenotype/outcome data did not (Table 5).

**Table 5.** Correlation of SF36v2 PCS and clinical outcomes and phenotype

​	Spearman’s ρ	P value
Age (years)	−0.370	*0.019*
Age (years) at diagnosis	−0.008	0.612
Annual infection incidence	−0.085	0.608
Latest FEV1 Z-score	0.178	0.356

Values in italics are those that are statistically significant (P <0.05).

There were no significant differences in median SF36v2 PCS for those receiving home IgRT versus hospital therapy (50.99 versus 48.14, P = 0.503). There were no significant differences in median SF36v2 PCS for those receiving IV versus SC IgRT (48.30 versus 51.50, P = 0.247).

#### St. George’s respiratory questionnaire (SGRQ)

A total of 43 adults filled out the SGRQ (Table 6).

**Table 6.** XLA SGRQ scores (median, IQR) compared those with and without bronchiectasis and against UK healthy norms (mean, 95% CI) (32) and UK CF patients (mean, SD) (33)

​	UK XLA patients (*n* = 43)	UK norms	P value	CF patients	P value
Symptom score	43.41 (14.32, 58.58)	12 (9–15)	*<0.001*	35.29 (19.3)	0.267
Activity score	18.47 (4.48, 35.61)	9 (7–12)	*<0.001*	28.90 (25.2)	0.412
Impact score	14.58 (4.58, 24.23)	2 (1–3)	*<0.001*	18.60 (14.6)	0.246
Total score	21.06 (6.37 – 27.86)	6 (5–7)	*<0.001*	24.50 (16.8)	0.156

​	Bronchiectasis	No bronchiectasis	P value	Bronchiectasis versus male UK norms	Bronchiectasis versus CF patients
Symptom score	56.33 (38.44, 68.26)	11.89 (0.00, 19.03)	*<0.001*	*<0.001*	*0.001*
Activity score	30.81 (18.47, 43.48)	1.16 (0.00, 5.96)	*<0.001*	*<0.001*	0.517
Impact score	21.66 (14.50, 31.39)	1.89 (0.00, 5.52)	*<0.001*	*<0.001*	0.139
Total score	26.84 (21.05, 32.94)	4.81 (1.52, 8.63)	*<0.001*	*<0.001*	0.198

Values in italics are those that are statistically significant (P <0.05).

UK XLA patients scored significantly worse in all domains of the SGRQ compared to UK norms, with comparable scores to CF patients.

XLA patients with bronchiectasis had significantly worse scores than patients without bronchiectasis in all domains of the SGRQ. Their scores remained significantly lower than UK norms and comparable to CF patients (Table 6).

A higher total SGRQ score correlated with increasing infection incidence, lower latest lung function results, and lower SF36v2 PCS (Table 7).

**Table 7.** SGRQ total scores and clinical outcomes and phenotype

​	Spearman’s ρ	P value
Age (years)	0.136	0.397
Age (years) at diagnosis	0.241	0.130
Annual infection incidence	0.419	*0.007*
Latest FEV1 Z-score	−0.426	*0.017*
SF36v2 PCS	−0.649	<0.001

Values in italics are those that are statistically significant (P <0.05).

For patients with bronchiectasis, there was no difference in the SGRQ total score between those on prophylactic antibiotics and those who were not (median 26.50 versus 27.30, P = 0.450). Azithromycin accounts for 52% of prophylactic antibiotics.

#### SF36v2 psychological health

UK XLA patients scored significantly higher in the mental role and mental health components of the SF36v2 compared to UK norms and significantly higher in the mental role domain compared to CF patients (Table 8).

**Table 8.** SF36v2 mental component scores (MCS) for the cohort (median, IQR), comparing those with and without bronchiectasis to UK norms (mean, SD) (30) and UK male patients with CF (mean, SD) (31)

Component	XLA patients (*n* = 42)	Male UK norms	P value	Male UK CF patients	P value
Energy/vitality	50.00 (37.50, 75.00)	60.81 (18.93)	0.170	62.2 (21.6)	0.170
Social functioning	100.00 (62.50, 100.00)	84.71 (22.56)	0.753	81.9 (22.5)	0.753
Role—mental	100.00 (91.67, 100.00)	88.08 (19.91)	*<0.001*	78.0 (35.6)	*<0.001*
Mental health	80.00 (65.00, 90.00)	74.32 (17.24)	*0.037*	75.5 (18.4)	0.222
MCS	51.87 (47.54, 57.16)	51.16 (9.34)	0.528	N/A	N/A

Component	Bronchiectasis	No bronchiectasis	P value	Bronchiectasis versus male UK norms	Bronchiectasis versus CF patients
Energy/vitality	43.75 (31.25, 50.00)	87.50 (62.50, 100.00)	*0.001*	*<0.001*	*<0.001*
Social functioning	75.00 (62.50, 100.00)	100.00 (100.00, 100.00)	*0.005*	0.126	0.126
Role—mental	100.00 (91.67, 100.00)	100.00 (91.67, 100.00)	0.695	*0.011*	*0.002*
Mental health	80.0 (65.00, 85.00)	95.00 (75.00, 100.00)	*0.013*	0.531	0.773
MCS	50.62 (45.85, 54.72)	59.89 (52.24, 62.47)	*0.009*	0.313	N/A

Values in italics are those that are statistically significant (P <0.05).

Patients with bronchiectasis scored significantly worse in all domains apart from the mental role compared to patients without bronchiectasis. UK XLA patients with bronchiectasis scored significantly lower in the energy/vitality and higher in the mental role domains compared to UK norms. Patients with bronchiectasis scored significantly lower in the energy/vitality domain and higher in the mental role domains compared to CF patients (Table 8).

There were no significant differences in median SF36v2 mental component score (MCS) for those receiving home IgRT versus hospital therapy (52.24 versus 49.68, P = 0.264). There were no significant differences in median SF36v2 MCS for those receiving IV versus SC IgRT (52.40 versus 50.50, P = 0.512).

SF36v2 MCS did not significantly correlate with measures of clinical outcome but did significantly correlate with SGRQ scores (Table 9).

**Table 9.** SF36v2 MCS correlation with clinical outcomes/phenotype

​	Spearman’s ρ	P value
Age (years)	0.161	0.321
Age (years) at diagnosis	−0.181	0.265
Annual infection incidence	−0.088	0.593
Latest FEV1 Z-score	0.166	0.389
SGRQ total score	−0.499	*0.001*

Values in italics are those that are statistically significant (P <0.05).

#### Hospital and anxiety depression scale (HADS)

32 adults filled out the HADS. UK XLA patients had comparable scores to UK male norms and CF patients in the HADS (Table 10).

**Table 10.** Clinical anxiety and depression categories and comparative HADS scores for the XLA cohort versus UK male norms (median, IQR) (34) and UK male CF patients (mean, SD) (35)

Clinical screening categories	Score	*n* (%)	95% CI
HADS—anxiety (possible)	8–10	6 (18.8%)	8.9–35.3%
HADS—anxiety (probable)	≥11	5 (15.6%)	6.9–31.8%
HADS—depression (possible)	≥8–10	2 (6.3%)	1.7–19.9%
HADS—depression (probable)	11	1 (3.1%)	0.5–15.7%

​	XLA patients (*n* = 32)	UK male norms	P value	UK male CF patients	P value
HADS—Anxiety score	5 (4, 9.5)	5 (2, 8)	0.147	5.7 (3.9)	0.561
HADS—Depression score	2.5 (0.5, 5.5)	3 (1, 6)	0.873	3.4 (3.3)	0.807

​	Bronchiectasis	No bronchiectasis	P value	Bronchiectasis versus UK norms	Bronchiectasis versus CF patients
HADS—Anxiety score	6 (5, 10)	4 (1, 7)	0.079	*0.024*	0.101
HADS—Depression score	4.5 (2, 7)	0 (0, 2)	*0.003*	0.066	0.122

Values in italics are those that are statistically significant (P <0.05).

#### Rosenberg self-esteem scale (RSES)

A total of 32 participants (aged 12 and over) completed the RSES questionnaire (Table S2). There was no association between RSES outcome and the presence of bronchiectasis. Total RSES scores were significantly lower for UK XLA patients compared to UK norms and CF patients.

## Discussion

By examining HRQoL in a large national cohort of XLA patients, these data are the first to demonstrate that the presence of lung disease is a major determining factor of HRQoL in people living with XLA. Patients with bronchiectasis had significantly worse scores in a number of HRQoL domains compared to UK healthy norms and comparable scores to CF patients and, in some domains, significantly worse scores than in CF.

There are major differences in HRQoL between those with and without bronchiectasis. In the absence of bronchiectasis, HRQoL is largely comparable to UK healthy norms. The correlation between lung function and a number of HRQoL measures demonstrates that improving respiratory health or reducing the risk of bronchiectasis should be a major driver in clinicians’ attempts to improve the HRQoL of XLA patients.

We also described the psychological impact of XLA on QoL. While screening scores for anxiety and depression were not significantly different than UK norms and CF patients, self-esteem scores were significantly lower. Scores in the depression component of the HADS were significantly lower in patients with bronchiectasis.

The results in this data correspond well with the recent large USA study on XLA QoL (36). Similar to our data, HRQoL for the overall adult USA cohort was not significantly lower than published norms, but increasing coexisting conditions did increase the number of domains that were significantly lower. However, these measures did not specially examine aspects of respiratory health. Similar to our study, the US team found no HRQoL difference in pediatric patients compared to norms. A likely reason for this may be the absence of bronchiectasis, which maybe a time-related risk and largely present in adulthood. This somewhat differs from data reported by Soresina et al., who found that in 25 pediatric XLA patients, the PedsQL 4.0 scores were lower than the background healthy population (23), although better than comparative children with rheumatic disease.

Data from this cohort examining clinical outcomes in XLA (26) demonstrate that rates of bronchiectasis remain high (40%) and are not affected by IgG trough levels, infection rates, or age at diagnosis of XLA. Only increasing age was associated with an increase in prevalence of bronchiectasis. These findings concur with other studies on lung disease in XLA (12, 37). While these data may be confounded by historical suboptimal care in older patients, they lend further evidence to concerns that rates of bronchiectasis (and other complications) will remain significant due to the limitations of current IgRT therapy. The clinical burden of bronchiectasis and its subsequent impact on HRQoL will likely remain unchanged unless significant advances are made to XLA management.

The role of prophylactic antibiotics, particularly macrolides like azithromycin, is well-established in reducing exacerbation frequency in non-CF bronchiectasis (38). In our cohort, while azithromycin was the predominant choice for prophylaxis, the use of prophylactic antibiotics was not associated with a significant improvement in SGRQ total scores among patients with established lung disease. This lack of difference in HRQoL may reflect the “real-world” clinical application of prophylaxis, where antibiotics are often initiated in patients with higher symptom burdens or more frequent flares. While these agents may be effective at decreasing infection rates, their impact on the daily physical and psychosocial dimensions of HRQoL may be tempered by the underlying structural lung damage already present in 60% of the adult XLA population (26, 38).

These data also show that, while the majority of XLA patients (70%) are on home IgRT, there are no significant differences in HRQoL in those receiving home versus hospital IgRT. While the convenience of home-based SC therapy is often preferred by patients, our data also demonstrate that the route of administration (IV versus SC) does not significantly influence physical or mental HRQoL scores. This suggests that the overall burden of XLA and its clinical complications, rather than the mode of immunoglobulin delivery, remains the primary driver of patient well-being. The decision to offer home or hospital IgRT is therefore likely to be a personalized decision and may change over time depending on personal circumstances. The ability to remain flexible in regard to IgRT services must be maintained by clinical services to able to offer patients the most appropriate IgRT route and service for them at that time. Efforts by national groups to ensure secure and ready supply of IgRT to patients with IEI must remain a priority (39).

CF was chosen as a comparator group, not only due its similarities in long-term evolving lung disease but also because CF outcomes have benefited greatly from significant investment and progress into research and therapies, namely that of newborn screening, cystic fibrosis transmembrane conductance regulator (CFTR) modulators, and gene therapy (40, 41). Although the long-term outcomes are yet to fully realized, it is possible that CF patients born today will have a better HRQoL than XLA patients born today. It should be noted the CF HRQoL comparator data used in this analysis (which is, at best, comparable to XLA patients and in some areas better) are taken from before either newborn screening or gene therapy entered routine clinical practice.

While our results identify bronchiectasis as a primary determinant of HRQoL, we acknowledge that less frequent complications, such as gastrointestinal (GI) disease and deafness, can be significantly debilitating for individual patients. Although the prevalence of these complications in our cohort was too low to ascertain a statistically significant impact on aggregate QoL scores, their presence underscores the complex, multisystem management required for XLA beyond IgRT.

These data potentially have implications for other primary antibody deficiencies (PADs) where the mainstay of treatment is IgRT rather than cure. It is possible that for these cohorts the limitations of IgRT and lack of cure may also result in high rates of complications such as bronchiectasis. HRQoL from PAD cohorts containing multiple individual IEIs do consistently demonstrate lower HRQoL in both pediatric and adult cohorts (24, 42, 43).

Our study has several limitations. Although it is one of the largest to specifically examine XLA patients, the study size is still small, and the cohort of 67 participants represents a partial (approximately one-third) representation of the estimated 200 XLA patients living in the UK (25). Furthermore, the cross-sectional design captures only a single point in time, which limits our ability to assess the longitudinal progression of lung disease and its evolving impact on HRQoL. As a national study involving multiple UK sites, we must acknowledge potential center-level practice variations that may influence management and patient reporting. Additionally, survivorship bias may be present; our adult cohort likely represents patients who have successfully navigated childhood complications, potentially underestimating disease severity in the wider population. The reliance on voluntary participation also introduces potential nonresponse bias, as patients with very poor health or those who are asymptomatic may be less likely to complete lengthy questionnaires. We also acknowledge era differences regarding our comparator datasets, noting that many healthy norms were established prior to more recent shifts in general population health expectations.

While we were able to examine the impact of bronchiectasis on QoL, rates of other complications (such as GI disease) were too small to ascertain their impact. Collaboration with international partners would help overcome this problem. While the HRQoL tools used in this study are widely used and validated in medical research they are not specifically designed nor validated in patients with IEI. Patients with IEI may have unique factors not covered/assessed by the current tools available, particularly coping with living with severe and rare diseases where knowledge regarding prognosis and treatment options is constantly evolving/changing resulting in a substantial amount of uncertainty for patients and families.

We acknowledge that while our results identify bronchiectasis as a primary determinant of HRQoL, several other factors may act as confounders. In our cohort, increasing age was significantly associated with both a higher prevalence of bronchiectasis and a decline in SF36v2 PCS. This suggests that age may independently influence physical health outcomes alongside chronic lung disease. Other potential confounders often cited in chronic immune deficiencies, such as socioeconomic status, the burden of therapy (e.g., home versus hospital IgRT), and the cumulative frequency of infections could also play a role. However, given the rarity of XLA and our relatively small adult cohort, it was not statistically feasible to perform a multivariable analysis to isolate these independent effects. Despite these limitations, the stark difference in HRQoL scores between those with and without bronchiectasis across multiple independent domains indicates that respiratory health remains a critical factor impacting patient well-being.

Given the expanding number of IEIs being discovered, an increased awareness of disease phenotype, an ever-increasing improving safety profile of curative HSCT, and the evolution of novel treatment options, HRQoL assessment must form a vital cornerstone of evaluating disease cohorts. This will help decide which patient and/or disease cohorts warrant consideration of changing treatment guidelines and which will need radical curative treatment options such as HSCT or gene therapy.

To improve the utility of HRQoL assessment in IEI, recent work has designed specific IEI HRQoL measurement tools (44). Further work is needed to design and promote validated QoL scoring tools for XLA and the wider IEI spectrum.

This study adds to the existing literature that bronchiectasis is the major determining factor of QoL in XLA. Without lung disease, it would appear that XLA patients could enjoy a normal QoL. As bronchiectasis may be inevitable due to the limitations in current therapies, it is possible that over time most patients with XLA will develop worse HRQoL scores than the healthy population. At best, comparable scores to CF patients may be achieved. The value of novel therapy strategies, namely advances in IgRT, HSCT, and gene therapy, urgently need to be reassessed specifically for XLA.

## Materials and methods

This was a cross-sectional analysis of HRQoL of UK XLA patients. The design of the study and choice of HRQoL instruments was influenced by our group’s previous pilot study (45).

Eligible patients were those who met the criteria for definitive diagnosis of XLA as per the European Society for Immunodeficiencies guidelines (46).

Ineligible patients were those who did not meet the criteria for a definite diagnosis for XLA, those who lacked capacity, or those who refused consent to partake in the study. Centers with potential eligible patients were identified through data provided by the UK Primary Immunodeficiency Network registry (https://www.ukpid-registry.co.uk/). Centers were invited to participate in the study and confirm eligible patients for study inclusion.

A favorable ethical opinion for the study was granted by the Tyne and Wear South NHS Research Ethics Committee (reference 16/NE/0268).

Clinical outcome data were also collected as part of this research project and are reported in a separate manuscript (26). Relevant outcomes from that analysis are presented here for analysis of correlation with HRQoL.

Bronchiectasis was defined as the presence of characteristic radiological features on the patient’s most recent HRCT chest scan. In accordance with routine clinical practice across participating centers, the indications for and timing of HRCT imaging were determined by local clinical teams based on clinical judgment. Diagnoses were based on official reports provided by local radiologists at each center. While specific scoring systems (such as airway-to-artery ratio or lack of tapering) were utilized at the discretion of the reporting radiologist, these data were not uniformly collected for this study. To ensure clinical relevance, HRQoL assessments were completed at the time of the patient’s last recorded follow-up, following the radiological diagnosis of bronchiectasis.

Data are presented as median and interquartile range (IQR). Some comparative population data and norms are published as mean (SD) where only mean values were available. Where this occurs, this will be clearly highlighted in the text. Given that means are provided, it can be safely assumed their data are normally distributed and the median value will lie closely to the mean. Data were compared against published norms and comparative population data using the one sample Wilcoxon signed-rank test. Spearman’s correlation was used to analyze correlations between continuous variables. To account for multiple testing, P values were adjusted using the Holm–Bonferroni method. Statistical analysis was performed using STATA v15.1 (https://www.stata.com/).

HRQoL scores were compared against UK norms and, where available, UK patients with CF. CF was chosen as a comparator given its similarities with XLA as a congenital disorder with lung disease as the major comorbidity and that current therapies do not aim to cure the underlying disease. As XLA is an X-linked recessive disorder, all participants in this study were male. Consequently, to ensure the most accurate comparisons, male-specific UK healthy norms and data from UK male CF cohorts were utilized for HRQoL assessments where such specific data where available.

The following HRQoL measures were used:

(1) SF36v2 (47)

The SF36v2 is a well-recognized tool for measuring the QoL in adolescents and adults and has been used extensively when researching patients with IEI and a range of other disease groups (48). It is a broad instrument to measure both physical and mental health; the PCS provides a consolidated measure of overall physical well-being and limitations (47). The SF36v2 was used for patients over 16 years of age. These data were compared against normative UK data (30) and for UK male patients with CF (31). The mean age of this CF cohort was 25.15 years, and the mean forced expiratory volume (FEV)1 percentage prediction was 55.6% (31). Lower scores indicate a lower HRQoL.

(2) PedsQL 4.0 (27)

The PedsQL 4.0 generic core scale questionnaire is a well-recognized self-reporting tool for measuring HRQoL (23, 49). This tool assesses health status across four domains; for example, the social functioning subscale measures a child’s ability to interact with peers and keep up with others their own age (27). It has been well validated and shown to be reliable and able to differentiate between healthy children and those with chronic diseases (49). It has been used previously to study the QoL in patients with PAD (24). There are modified versions of the questionnaire for children and young people, depending on their age and a separate one for parents. The questionnaire was given to children aged 5–16 and parents of children aged 2–16. The data were compared against UK population norms (27) and patients with CF (male and female) (28). The age range of this CF cohort was 2–18 years, with a mean FEV1 percentage predicted of 88.5% (28). Lower scores indicate a lower HRQoL.

(3) The SGRQ (32)

The SGRQ is a widely used tool for recording HRQoL regarding respiratory health in adults, measuring the impact of chronic respiratory symptoms and (e.g., cough and wheeze) associated activity limitations on daily life (32). It was initially designed for chronic obstructive pulmonary disease and asthma but has since been well validated in bronchiectasis (50), including a direct correlation with mortality (51). It has also been shown to be reliable studying patients with PAD (52). These data were compared against UK healthy norms data (32) and patients with CF (male and female) (33). The mean age of this CF cohort was 24.50 years, and the mean FEV1 percentage predicted was 63.6% (33). Higher scores indicate a lower HRQoL.

(4) SDQ (53)

The SDQ was used for children aged 4–16 years old. The SDQ is a well-recognized measure of social, emotional, and behavioral difficulties in children and is a widely used tool for screening for psychological difficulties in childhood (53). It scores across several domains; for example, the peer problems score specifically evaluates difficulties with friendships, social isolation, or bullying (53). It has also been used in studies of patients with IEI (24). There are both self-reporting and parent scores. These were compared against published normative UK data (29). Higher SDQ scores indicate more difficulties in the areas measured.

(5) HADS

The HADS is 14-item scale screens for symptoms of anxiety and depression while intentionally excluding somatic symptoms that could be confused with medical illness and is validated in both adolescents and adults (12 years and over) (54). It can be used to both screen for disease and to assess severity. Higher scores indicate more symptoms associated with anxiety and depression. Raw HADSs were compared against UK male norms (34) and UK male CF patients (35). The mean FEV1 percentage predicted for this CF cohort was 61.6% (35).

(6) RSES (55)

The RSES is a 10-item scale widely used in clinical research to ascertain self-esteem (55). The RSES was used in participants aged 12 and upward. Raw scores were compared against UK male CF patients (56) and UK normative male data (57). The mean age of the UK male CF patients was 27.8 years, with a mean FEV1 percentage predicted of 61.7% (57). A higher score indicates a higher self-esteem.

### Online supplemental material

Table S1 displays the likelihood of emotional, conduct, and hyperactivity disorders from the SDQ in the UK XLA cohort. Table S2 shows RSES outcomes in the UK XLA cohort versus UK norms and CF patients.

## Ethics statement

All participants and/or their carers/parents provided informed consent, and a favorable ethical opinion for the study was granted by the Tyne and Wear South NHS Research Ethics Committee (reference 16/NE/0268).

## Supplementary Material

Table S1 shows likelihood of emotional, conduct, and hyperactivity disorders on the SDQ.

Table S2 shows RSES outcomes and comparison (median, IQR) versus UK norms (mean, SD) (57) and CF patients (mean, SD) (56).

## Data Availability

All relevant data that can be shared can be found in supplemental material. Additional data are available from the corresponding author upon reasonable request from health care professionals and within national General Data Protection Regulation rules and regulations.
