# Supplementary material for: Quality of life of X-linked agammaglobulinemia patients in the United Kingdom
Source: J Hum Immun. 2026 Feb 19;2(3):e20250198. doi: 10.70962/jhi.20250198 (PMC13177391; doi:10.70962/jhi.20250198)
Supplement: Table S1 — shows likelihood of emotional, conduct, and hyperactivity disorders on the SDQ. [file jhi_20250198_tables1.docx]

Supplemental table 1 Likelihood of emotional, conduct and hyperactivity disorders on the SDQ

|  | **Category** | **n (%)** | **95% Confidence Interval (CI)** |
| --- | --- | --- | --- |
| **Hyperactivity Disorder** | Unlikely | 19 (79%) | 59.5% – 90.9% |
|  | Possible | 5 (21%) | 9.2% – 40.5% |
|  | Probable | 0 (0%) | 0.0% – 14.3% |
| **Conduct Disorder** | Unlikely | 16 (67%) | 47.1% – 82.1% |
|  | Possible | 6 (25%) | 11.9% – 44.9% |
|  | Probable | 2 (9%) | 2.3% – 25.8% |
| **Emotional Disorder** | Unlikely | 19 (79%) | 59.5% – 90.9% |
|  | Possible | 4 (17%) | 6.7% – 35.9% |
|  | Probable | 1 (4%) | 0.7% – 20.8% |
