# Supplementary material for: Quality of life of X-linked agammaglobulinemia patients in the United Kingdom
Source: J Hum Immun. 2026 Feb 19;2(3):e20250198. doi: 10.70962/jhi.20250198 (PMC13177391; doi:10.70962/jhi.20250198)
Supplement: Table S2 — shows RSES outcomes and comparison (median, IQR) versus UK norms (mean, SD) (57) and CF patients (mean, SD) (56). [file jhi_20250198_tables2.docx]

Supplemental Table 2 RSES Outcomes and comparison (median, IQR) versus UK norms (mean, SD) (57) and CF patients (mean, SD) (56).

| RSES outcome | | Number of patients (n, %) | | | 995% Confidence Internal (CI) |
| --- | --- | --- | --- | --- | --- |
| Low self-esteem | | 5, 16% | | | 0.0% - 30.1% |
| Normal self-esteem | | 11, 34% | | | 3.9% - 68.4% |
| High self-esteem | | 16, 50% | | | 31.6% - 86.1% |
|  | | | | | |
| RSES outcome | | **Bronchiectasis (n = 18)** | | | **No bronchiectasis (n = 9)** |
| Low self-esteem | | 5, 28% | | | 0, 0% |
| Normal self-esteem | | 5 28% | | | 5, 38% |
| High self-esteem | | 8, 44% | | | 8, 62% |
|  | | | | | |
| XLA (median, IOR) | **UK Norms**  **(n = 32)** | | **p value** | **CF patients** | **p value** |
| 25.50 (19.50, 29) | 31.68 (5.67) | | **<0.001** | 33.94 (5.1) | **<0.001** |
